# “How long is life worth living for the horse?” A focus group study on how Austrian equine stakeholders assess quality of life for chronically ill or old horses

**DOI:** 10.1186/s12917-024-04211-8

**Published:** 2024-08-06

**Authors:** Mariessa Long, Herwig Grimm, Florien Jenner, Jessika-M. V. Cavalleri, Svenja Springer

**Affiliations:** 1 Messerli Research Institute, Department of Interdisciplinary Life Sciences, University of Veterinary Medicine, Vienna, Medical University of Vienna, University of Vienna, Veterinaerplatz 1, 1210 Vienna, Austria; 2 Equine Surgery Unit, Clinical Centre for Equine Health and Research, Clinical Department for Small Animals and Horses, University of Veterinary Medicine, Vienna, Veterinaerplatz 1, 1210 Vienna, Austria; 3 Equine Internal Medicine Unit, Clinical Centre for Equine Health and Research, Clinical Department for Small Animals and Horses, University of Veterinary Medicine, Vienna, Veterinaerplatz 1, 1210 Vienna, Austria

**Keywords:** Horse welfare, Quality of life, End-of-life decisions, Euthanasia, Veterinary care decisions, A good life

## Abstract

**Background:**

Quality of life (QoL) provides a comprehensive concept underpinning veterinary decision-making that encompasses factors beyond physical health. It becomes particularly pertinent when seeking responsible choices for chronically ill or old horses that emphasise their well-being and a good QoL over the extension of life. How different stakeholders use the concept of QoL is highly relevant when considering the complexity of these decisions in real-life situations.

**Methods:**

Seven focus group discussions (*N* = 39) were conducted to gain insights into how stakeholders assess and use equine QoL in veterinary care decisions for chronically ill and/or old horses. The discussions included horse owners (*n* = 17), equine veterinarians (*n* = 7), veterinary officers (*n* = 6), farriers (*n* = 4), and horse caregivers (*n* = 5). The combination of deductive and inductive qualitative content analysis of the group discussions focused on identifying both similarities and differences in the views of these groups regarding QoL for old and/or chronically ill horses.

**Results:**

Findings show agreement about two issues: the importance of the individuality of the horse for assessing QoL and the relevance of QoL in making decisions about veterinary interventions. We identified differences between the groups with respect to three issues: the time required to assess QoL, stakeholders’ contributions to QoL assessments, and challenges resulting from those contributions. While owners and caregivers of horses emphasised their knowledge of a horse and the relevance of the time they spend with their horse, the veterinarians in the study focused on the differences between their own QoL assessments and those of horse owners. In response to challenges regarding QoL assessments and decision-making, stakeholders described different strategies such as drawing comparisons to human experiences.

**Conclusions:**

Differences between stakeholders regarding equine QoL assessments contribute to challenges when making decisions about the care of chronically ill or old horses. The results of this study suggest that individual and collaborative reflection about a horse’s QoL should be encouraged, for example by developing practicable QoL assessment tools that support relevant stakeholders in this process.

**Supplementary Information:**

The online version contains supplementary material available at 10.1186/s12917-024-04211-8.

## Background

Equine medicine offers increasingly advanced diagnostic and therapeutic options for treating horses and, if possible, prolonging their lives. Whereas in the past horses were used mainly for work purposes, such as in agriculture, in Western societies they are today increasingly kept as companion animals for leisure use [1, 2]. The combination of the resulting close emotional bonds between owners and their horses and the increasing and advanced options of equine care can lead to challenging questions about end-of-life treatment for chronically ill or old horses [3–6]. In such situations, the focus changes from what is medically feasible to what constitutes the ethical and responsible course of action for an individual horse. In relation to this, the concept of the horse’s quality of life (QoL) brings relevant factors that go beyond mere physical health into veterinary decisions and actions. QoL plays an important role in horse owners’ decisions about veterinary treatment for their geriatric horses [7], especially for decisions regarding euthanasia of their horse [3, 5, 7–10].

Although debates about the differences and similarities in the concepts of animal welfare and QoL are ongoing [11–15], explicit definitions for QoL have been developed. For instance, Belshaw and Yeates [16] state that QoL “represents the aspects of an animal’s life that make that life better or worse for that specific animal.” Taylor and Mills [17] define animal QoL as “the state of an individual animal’s life as perceived by them at any one point in time. It is experienced as a sense of well-being which involves the balance between negative and positive affective states and any cognitive evaluation of these, where the animal has the capacity.” Yeates [15] points out that in contrast to welfare, “QOL is essentially considered over a longer interval.” When these definitions are used, decisions about veterinary care based on a horse’s QoL require the inclusion of facets of a horse’s life that expand the focus beyond the sole consideration of the horse’s physical health. This entails evaluating all relevant aspects of a horse’s life, even those that are challenging to assess, such as the horse’s emotional state [18]. When going as far as considering the horse’s experience across their lifetime the goal is a continuously high QoL resulting in an overall ‘Good Life’, a concept also recognised and advocated for in equestrian contexts [19]. When dealing with chronically ill horses, focusing only on illness disregards how the illness affects the horse and how the horse’s QoL can be enhanced even when a cure is unattainable. Thus, the concept of QoL becomes particularly pertinent for horses dealing with chronic conditions as a result of ageing or other factors.

A recurring topic in studies exploring horses’ well-being, QoL and welfare is the tension between knowing the horse well but staying detached and sufficiently objective to assess the horse’s QoL and make decisions about veterinary care [4, 20–22]. Horse owners play a key role in this, as they decide about the day-to-day care and management of their animals. Horse owners’ interpretations of a horse’s well-being and subsequent decision-making are influenced by various factors related to the relationship between an individual horse and their owner [23, 24]. These include past experiences with the horse, the horse’s intended purpose, the horse’s physical responses, and the presumed subjective experience of the horse [23, 24]. Horse owners keep their horses for different purposes and in different contexts [25, 26], and it is known that they can develop strong emotional bonds with their horses [5, 26, 27]. This emotional bond has positive implications for the horse’s QoL because it motivates owners to strive for patient-centred decisions and allocate resources towards the care of ageing or chronically ill horses [6, 27]. According to horse owners, knowing the horse provides them with important information for assessments of health and QoL [24, 25]. On the other hand, a close relationship with a horse combined with a lack of expertise can also conflict with effective and appropriate long-term care. For instance, horse owners were found to struggle with taking necessary measures to reduce the weight of their horses because they perceived the measures to conflict with the horse’s short-term well-being, their beliefs about appropriate horse care, and the relationship they had with their horse [28]. Horse owners acknowledged the emotional difficulties they experienced when making a decision to euthanise their horse [5] which can negatively affect the horse when the decision is drawn out. In relation to this, delayed euthanasia has been identified as a major welfare concern for horses [29–32]. In addition, horse owners do not always recognise the significance of health issues in their geriatric horses [33–35] and can misinterpret or underestimate behavioural indicators of a horse’s distress [36, 37]. In summary, an owner’s close relationship with a horse can be beneficial for QoL assessments because of their knowledge of a horse and their interest in the horse’s well-being. At the same time, horse owners may lack expertise and be biased against recognising their horse’s QoL issues.

Horse owners frequently point to veterinary advice as a crucial influence when making decisions regarding general veterinary care and euthanasia for their horses [3, 5–9, 26, 27, 38]. The relationship between a veterinarian and an equine patient is different from that between an owner and their horse. Veterinarians spend less time with their patients, but their assessments of a horse’s health and QoL are also less likely to be influenced by emotions. Instead, veterinarians encounter a horse patient in their capacity as an expert on horse health and disease. Financial concerns can complicate the situation for veterinarians, especially when clients are unable or unwilling to pay for necessary treatments for their animals [39, 40]. Veterinary officers, who can be called in by veterinarians and are tasked with enforcing animal welfare laws, are likely to encounter more extreme cases, such as severely neglected horses. This has the potential to affect how they evaluate equine QoL and the aspects they deem relevant. How their assessment of QoL compares with that of a general equine practitioner is thus a topic of interest.

Although horse owners and veterinarians are undoubtedly central stakeholders when assessing QoL to make decisions for chronically ill or old horses, other stakeholders can directly and indirectly contribute to QoL assessments for animals. Horse owners’ perceptions of appropriate horse care are influenced by the views of people in their social circles [24, 41], such as other horse owners and horse caregivers working in a stable. Horse caregivers might also be involved in directly monitoring and assessing the QoL of old or chronically ill horses by offering an additional perspective. Farriers are also a relevant source of information for horse enthusiasts [26]. Because farriers encounter a horse on a regular basis, they might contribute to QoL assessments, for example by noticing changes in a horse’s condition or behaviour. To understand how these different stakeholders influence decisions for chronically ill or old horses, it is important to gain insights into their perspectives on QoL assessment and how they use the concept in the context of decisions about veterinary care.

No designated equine QoL protocols are available to help horse owners and caregivers of chronically ill and/or old horses. The horse welfare tools that exist would need adjustments to make them useful in the context of decisions about the care of such horses [42]. More generally, horse owners and other stakeholders do not use formal assessment protocols for equine well-being but instead rely on informal evaluations [20, 24]. The subjective nature of the concept of QoL is likely to make its assessment and prediction as a basis for decisions about veterinary care challenging [43].

Existing qualitative studies on equine well-being assessment with multiple equine stakeholders have focused on horse welfare or happiness in different contexts but have not yet explored how equine stakeholders use and understand equine QoL particularly for old or chronically ill horses [20, 21, 26, 32, 44–46]. The present study aimed to empirically investigate the assessments of equine QoL of a variety of stakeholders including equine veterinarians, veterinary officers, horse owners, farriers, and horse caregivers in the context of making decisions about veterinary care for chronically ill or old horses. We interviewed these different stakeholder groups in a focus group study regarding the role of QoL in decision-making about veterinary care, QoL assessments, and the challenges associated with assessing equine QoL in order to identify similarities and differences in the views of these groups.

In this study, our explicit focus was on QoL because of the negative connotation of the term ‘welfare’ and its possible association with severe cases of neglect among some equine stakeholders [21, 32] and the likely association of German translations of welfare (e.g. “Tierwohl”) with animal welfare standards in farming instead of veterinary care for individual horses. Furthermore, other terms, such as QoL, are favoured by stakeholders in practice [20]. UK leisure horse owners used different terms to identify well-being, sometimes interchangeably, and showed a tacit understanding of how each term was meant [24]. However, QoL was mostly used in relation to the positive aspects of a horse’s life and in relation to decisions about euthanasia [24], both of which are relevant in the context of making decisions about veterinary care for chronically ill or old horses. Therefore, this study focused on and specifically asked participants about equine QoL. Because of the various stakeholders’ different relationships with horses varying from professional to highly emotional, we expected that evaluating QoL can be a challenge and a source of disagreement among stakeholders in the context of decisions about veterinary interventions for chronically ill and old horses.

The aim of the study was to gain insights into how horse owners, equine veterinarians, veterinary officers, farriers, and horse caregivers assess and use equine QoL. We addressed three research questions: (1) How do horse owners, equine veterinarians, veterinary officers, farriers, and horse caregivers assess QoL, particularly for chronically ill or old horses? (2) What role does the QoL of old or chronically ill horses play in making decisions about veterinary care? (3) What challenges do these various stakeholder groups face in relation to the assessment of QoL of old or chronically ill horses and how do they deal with them? Throughout our analysis, we focused on differences and similarities among the stakeholder groups.

## Methods

### Recruitment process

We recruited equine veterinarians, veterinary officers, owners of chronically ill or old horses, horse caregivers, and farriers for the focus group study via several means and with different approaches for different groups (for details about each approach see Additional File 1). An advertisement for the study was posted on the Facebook page of the University of Veterinary Medicine, Vienna, on 3 June 2021 that invited participants from all groups, namely equine veterinarians, veterinary officers, horse owners with chronically ill horses or horses older than 19 years, horse caregivers, and farriers. In addition to basic information about the mode of the study, the invitation featured the question “How can good decisions be made for chronically ill and old horses?” We also sent both personalised and general invitations using a variety of methods (see Additional File 1). In all stakeholder groups, recipients of invitations were encouraged to pass the invitation on to others who might be interested in the study. Participants were offered €50 compensation for participating in the focus group study, which all but one participant accepted.

According to a horse’s physiological or demographic age horses are generally classified as old when 15 years or older [47]. However, as we were interested in equine stakeholders’ practice and perception, we chose to classify horses as old when 20 years or older to reflect findings showing that horse owners tend to consider their horse old at the age of 22 on average [48] and at the distinct age of 20 or 25 years [49]. We asked participants during the recruitment process whether their horse(s) had a chronic illness that could presumably not be cured anymore.

Before the study, we provided information to all participants about the type of data that would be recorded in the study and how those data would be handled. We also informed them that participation was voluntary and that they were free to withdraw their consent at any time during or after the study. All participants provided written consent and no participant withdrew their consent during or after the study.

### Study participants

Seven focus group discussions with a total of 39 participants living in Austria were conducted from June to November 2021. The number of participants per group ranged from 4 to 7 and the composition of the groups was homogenous regarding the inclusion criteria as presented in Table 1. To enhance readability, we have categorised the participants in the results section as either veterinary professionals (equine veterinarians and veterinary officers) or non-veterinary participants (horse owners, horse caregivers, and farriers).

**Table 1:** Characteristics of focus groups and participants

#	Group	Inclusion criteria	Other details	*N*	Female	Male	Federal state^*^
**1**	Equine veterinarians	Min. 50% of work time dedicated to horses or trained as equine specialist	All owned at least 1 horse	7	6	1	T, LA, UA
**2**	Veterinary officers	Veterinarian employed as a veterinary officer by an Austrian authority	3 owned at least 1 horse	6	4	2	LA, UA, ST, V
**3**	Farriers	Certified training as a farrier or min. 5 years of work experience as farrier	3 owned at least 1 horse	4	1	3	LA, UA, V
**4**	Horse caregivers	Certified training as horse caregiver or animal keeper or min. 2 years of experience as an employed horse caregiver	3 owned at least 1 horse; 2 worked in a horse clinic; 2 worked at a sanctuary; 1 was a stable owner	5	5	0	V, LA, SB
**5**	Owners of horses for leisure use	Own min. 1 horse older than 19 years and/or a chronically ill horse (or owned one within the past 6 months); no professional or commercial use of horses		7	7	0	V, UA, ST, K, LA,
**6**	Owners of horses for income	Own min. 1 horse older than 19 years and/or a chronically ill horse; owns horses for professional or commercial use to generate income (or owned one within the past 6 months)	1 stable owner and riding instructor; 2 riding instructors and horse trainers; 1 riding school and stable owner	4	4	0	K, LA, V
**7**	Owners of horses for leisure and sanctuary owners	Own min. 3 horses older than 19 years and/or a chronically ill horse as part of an animal sanctuary or shelter		1	1	0	B, UA, LA, V
		Own min. 1 horse older than 19 years and/or a chronically ill horse (or owned one within the past 6 months)	3 leisure horse owners; 1 hobby horse stable owner; 1 veterinary student	5	5	0	
**Totals**				39	33	6	B, UA, LA, V, K, ST, SB, T

^*^B: Burgenland; LA: Lower Austria; K: Carinthia; SB: Salzburg; ST: Styria; T: Tyrol; UA: Upper Austria; V: Vienna.

### Procedure and structure of focus group discussions

The focus group discussions were conducted online due to the unpredictability of the situation during the COVID-19 pandemic. In order to capture spontaneous replies by participants and keep the discussion as close as possible to an in-person focus group discussion, the discussions were conducted via synchronous videoconferencing [50], a strategy that other research groups have used successfully [51, 52]. Research that compared the data gained through focus group discussions via synchronous videoconferencing with data from in-person focus group discussions shows similar engagement in the discussion and a similar quality of the resulting data [53].

The focus group discussions were conducted in German using the web conferencing tool Cisco Webex (Cisco Systems, Inc., San Jose, USA). All focus group discussions were videorecorded using this tool. The participants were encouraged to turn on their cameras, which all of them did except for one participant who had technical issues. The duration of the sessions ranged from 140 to 150 min; the average was 146 min.

The focus group discussions were hosted by a moderator (HG) and a second person from the project team (ML), who took notes, provided instructions on time limits, and typed comments to the moderator via direct chat messages that were not visible to the participants. ML was also available to help with technical issues. The most important questions from the interview guide were shared with the participants via a presentation during the discussion.

Prior to the focus groups, the interview guide was piloted twice to ensure understandability and feasibility, once with a horse owner and once with a veterinarian and a veterinary student who was also a horse owner. Their feedback and comments were integrated into the final version of the interview guide.

The interview guide for the semi-structured discussions was developed based on literature on QoL and welfare (see [42] for a review) and with consideration of our research questions and expectations for the study. The interview guide was separated into four parts and was structured using a funnel approach: questions started at a general level and became successively more specific. A detailed presentation of the structure of the focus group discussions can be found in Additional File 2. Part one of the interview guide included a brief introduction to the study. Participants were informed that more details about the content of the study would not be shared until after the discussion to minimise any influence on the results. Subsequently, the moderator (HG) and the second researcher (ML) introduced themselves. After that, we asked group members to answer specific questions as they introduced themselves. All participants were asked to talk about their experience with horses, but we also asked them to cover specific issues based on their relationship to horses. This first part of the group interviews took an average of 16 min; the range was 12 to 22 min.

Part two of the interview guide addressed the information that participants required for assessing a horse’s QoL. The average duration of this part of the group interview was 58 min; the range was 42 to 70 min. Our goal was to encourage discussion within the groups and to motivate participants to give reasons for why they considered certain aspects important for assessing a horse’s QoL. We did not seek to arrive at an overall ranking for each group.

In part three of the interview guide, participants were invited to share their experiences with difficult decisions about veterinary treatments for their own horses (in the case of horse owners) or horses they had encountered in their professional roles for all other categories of participants. The average duration of this part of the interview was 25 min; the range was 22 to 29 min.

Lastly, part four of the interview guide provided two fictional cases, one about a blind horse and one about an overweight horse. The average duration of this section was 28 min; the range was 28 to 38 min. We invited participants to share their perspective on the QoL of these two horses to identify potential differences and similarities between individuals and groups and to identify how the different stakeholders arrived at a conclusion about the horses’ QoL in the fictional cases.

Owing to technical issues, the recording of one of the focus groups (Group 5, horse owners) was cut short midway through part three of the interview guide (at 11 min of 22 min of part three), when discussing the horse owners’ experiences with difficult decisions. The rest of the session, including participants’ assessments of the fictional cases could not be included in the analysis for this group.

### Data analysis

We studied our participants’ accounts of their perspective on equine QoL in the context of focus group discussions that encourage interaction and exchange between the participants. We acknowledge the influence of the researcher as an active part of the knowledge production and refer to Additional File 3 for a reflexivity statement and details on the research project team members’ backgrounds.

Video recordings have been transcribed verbatim, following the guidelines for content-semantic transcription [54]. They have been coded using the MAXQDA 2020 (v20.4.2) software program (VERBI – Software. Consult. Sozialforschung. GmbH, Berlin, Germany). Analysis was performed on the German transcript. The quoted statements in the results were translated into English by ML, preserving their original wording and meaning as much as possible.

Two coding cycles were used to analyse the data through qualitative content analysis [55]. Following a template organising style [56], the first coding cycle used deductive (mainly descriptive) hypothesis and holistic coding [55]. This was done to organise the data units in accordance with categories and codes developed based on literature on animal QoL and welfare to account for existing theory, research questions, expectations and the interview guide [57]. Two authors (ML and SS) used the initial coding list, which contained 25 codes in four categories, to independently code the same focus group transcript. Following a discussion of coding results, the code list was adjusted to remove, adjust, and clarify individual codes. This resulted in three categories with a total of 18 codes. ML then coded the transcripts of all focus group discussions using the adjusted code list. The second coding cycle used an inductive approach to identify additional codes and patterns within and across categories and existing codes to allow for an analysis beyond existing theory [57]. This resulted in a final code list of 22 codes in three categories (see Additional File 4).

Combining a deductive and an inductive approach to coding allowed for a structured comparison of the groups based on the final code list. The final code list provided the basis for initial analysis of the coded segments in individual groups before groups were compared against each other. Through summaries of the coded segments the coder (ML) integrated coded segments into broader categories which were then tested against the original data. This took place in discussion with SS and with a focus on similarities and differences within and between groups. In addition, findings and selected segments of data were continuously discussed within the project team (ML, SS, HG, FJ). The analysis was based on anonymised transcripts. The original video recordings were consulted by the coder (ML) only if it was necessary to resolve uncertainties with regard to the meaning of the transcribed text (e.g., listening to see if the participant emphasised certain words).

## Results

The study results provide important insights into how various stakeholder groups conceive of and use the concept of QoL in relation to old and chronically ill horses. First, we identified insights into issues that are important for QoL assessment, namely the individuality of the horse, the time required to assess equine QoL and contributions from different stakeholders. Second, this study reveals insights into the role of QoL in decision-making processes regarding veterinary treatments. Finally, we identified challenges related to the horse or to other stakeholders during QoL assessments and decision-making and strategies for dealing with these challenges.

### Important aspects for the assessment of QoL

From the focus group discussions, we identified three issues as central in the assessment of a horse’s QoL: the individuality of the horse, the time required to assess equine QoL, and contributions from various stakeholders.

#### The individuality of the horse

Throughout all focus group discussions, we identified the individuality of the horse as the fundamental issue regarding assessments of equine QoL. This included the question of whether a horse was living under conditions that were suitable for that individual. For example, one horse owner explained that “[…] you can say that in principle, according to my feeling, the husbandry conditions would be OK for a horse, but I don’t know if that now also applies to this one [particular] horse […]” (FG5-own7). Similarly, an equine veterinarian stated that it is “[…] always an individual decision. With one horse one point may weigh more and with another horse the other point weighs more.” (FG1-vet3).

Because the individual horse and the fit with the horse’s living context were so important in QoL assessment, participants in all groups emphasised that when assessing a horse’s QoL it was important to consider a lot of information about the horse, the “whole package” (FG5-own1, FG6-own2), as two horse owners summarised it. One horse owner said that it was necessary to get “[…] an overall picture. What does the horse do all day and what are the internal and external factors that apply to [the horse] in that case?” (FG5-own2).

Although some participants in the farrier and horse owner groups reported that answers to rather broad questions about a horse would be sufficient to give an estimate of a horse’s QoL, the majority of participants across the groups described different important forms of contact with horses to assess QoL. This included especially seeing the horse, but participants also mentioned tactile and olfactory contact, interaction and the flow of energy. For example, one equine veterinarian pointed out that “[…] three questions are not enough, especially just listed facts are not enough. So you have to definitely see the horse […]” (FG1-vet2). In the words of another equine veterinarian, “You get the information directly from the living being.” (FG1-vet5).

#### Time required to assess QoL

We identified differences of opinion between the groups with respect to the time needed to assess a horse’s QoL. Many participants stated that it was important to assess a horse over a “longer period of time” (FG5-own4, FG7-own2, FG7-own3, FG4-care5). This notion was particularly strong in the non-veterinary groups. For instance, one horse owner stated that it “[…] is very difficult for a therapist or a veterinarian to judge, because they only ever have a snapshot.” (FG5-own5).

Even though veterinarians acknowledged that seeing a horse over a longer time or in different contexts can be beneficial, they reported that they were able to make a quick initial assessment based on their “first impression” (FG1-vet7) and “feeling” (FG1-vet7) about the horse and the horse’s environment. For example, one equine veterinarian said, “Those are all things, little things, that are part of it, that one – I have also been in this job for more than 20 years by now – perceives. That happens quickly.” (FG1-vet5). Furthermore, veterinary officers in particular pointed towards the importance of regular, repeated veterinary check-ups, especially in the context of decisions about treatments or euthanasia for chronically ill horses. A veterinary officer recounted a “borderline case” where “[…] you then also really have to be on site several times, in order to see, is the animal’s quality of life really still there. Something like that requires frequent monitoring, that you possibly go there more often and take a look, whether [continued treatment] is justifiable.” (FG2-offvet1).

#### Contributions to QoL assessments from different stakeholders

Horse owners often pointed out that they knew their horse well and that this gave them an advantage when evaluating the horse’s QoL, including the ability to detect subtle changes. One horse owner, for example, reported of her chronically ill horse, “If, of course, a stranger sees this, they will think, good heavens, what am I doing with the horse? That’s animal cruelty! I, however, have known the horse since it was a foal, he was born here with me and I know [that] he is happy with it.” (FG5-own1). Horse caregivers also emphasised the relevance of knowing a horse well. However, the opinions among horse owners were not uniform and ranged from seeing themselves as the experts regarding their horse to seeing themselves and other horse owners as biased in their assessments of the QoL of their horses even if they wanted the best for their animal. Equine veterinarians and veterinary officers agreed that bias can creep into horse owners’ assessments. They said that compared with many horse owners and people without veterinary training, their expertise and experience enabled them to more accurately identify and assess problems related to a horse’s QoL.

Although horse owners portrayed their own input as very important when assessing the QoL of their horse, they also recognised the importance of veterinary input and professional expertise regarding the health and illness of a horse. One horse owner described “parameters on paper” (FG5-own5) such as blood test results and X-Ray images as “[…] one puzzle piece of many to get an overall impression.” (FG5-own5). Another horse owner described her veterinarian as a “partner” (FG6-own2) when determining the state of her horse. A horse owner summed it up by describing the horse owner—that is, the person who spends the most time with the horse—as the expert “[…] in terms of the horse’s emotions and feelings […]” but conceded that regarding the health status of the horse it is probably the veterinarian “[…] who knows better in the end” (FG7-own5).

Whereas the findings of this study show that horse owners and equine veterinarians played central roles in equine QoL assessment, horse caregivers and farriers described their own contributions as less central. For horse caregivers, this varied with the context they worked in. The closer their relationship with a horse came to that of a horse owner, the more relevant their role became in QoL assessments, for example in a horse sanctuary. Although farriers acknowledged that because they see a horse regularly, they could sometimes recognise changes in the horse’s behaviour, most rejected an overall assessment of a horse’s QoL in their role as a farrier and emphasised instead that their expertise and influence related to the treatment of horses’ hooves. One farrier described their collaboration with equine veterinarians as part of a “veterinarian–farrier team” in which he regards the veterinarian as the “higher authority” (FG3-far3).

### The role of QoL in decision-making about veterinary interventions

We identified that equine QoL plays an important role in making decisions about veterinary interventions in three ways: (1) the horse’s current QoL; (2) the predicted QoL during and after treatments; and (3) the improved QoL after treatments.

First, the horse’s current QoL was used as a justification for deciding to pursue or not pursue treatment or management changes for a horse. Participants in different groups often referred to a horse’s good QoL or the lack thereof as a reason to intervene or not intervene. For instance, an equine veterinarian said that when there is “[…] no longer a quality of life […] I am really in favour of putting it to sleep.” (FG1-vet4). Good QoL was identified as a factor in deciding against euthanasia. For example, a horse owner argued that when “[…] the quality of life is nevertheless very good […] I don’t actually see any reason why I should have to make any decisions there [about] whether I would maybe have to let him go for such-and-such a reason.” (FG7-own3). Participants also discussed QoL as gradual in the sense that it could be better or worse rather than as something that was entirely present or absent.

The absence of a horse’s will to live was cited in discussing euthanasia decisions, particularly in the non-veterinary groups. For example, a horse caregiver explained, “I mean, you can see it in their eyes or in their overall condition, whether they simply want to go now or whether we try [treatment] again.” (FG4-care5). However, the horse’s will to live was not always enough to decide against euthanasia. For example, one horse owner said that “[…] although the horse itself was not mentally ready to lie down to die […]” (FG6-own1), they justified the decision to euthanise because of the horse’s condition and because “there would not have been a quality of life anymore” (FG6-own1).

Second, the predicted QoL during and after treatments was an important factor when choices between several types of treatments had to be made. A veterinary officer explained that “[…] of course you can treat a lot for a relatively long time, [but] the question is always how much does it affect the quality of life of the horse?” (FG2-offvet2).

Third, QoL played a role in justifying past decisions when these decisions had led to an improved QoL for the horse. Although the opinion of a veterinarian was generally presented as important and influential in decisions about medical care, some horse owners also recalled experiences where they had decided against following a veterinarian’s recommendation for euthanasia. In discussing these cases, participants said that an improved QoL showed that their decision to continue a horse’s treatment had been justified. There were also instances of participants describing outcomes of veterinary interventions as justifying their decisions to continue treatment when this had not been opposed to a veterinarian’s advice.

Results pointed to factors in decisions about treatments that had the potential to outweigh considerations of the horse’s QoL. Veterinary professionals and horse owners described other horse owners to be influenced by the owner’s emotional attachment to the horse, the previous or intended use of the horse, and the owners’ financial and management resources. Veterinary officers also cited legal frameworks that guided their actions; these frameworks sometimes required them to make decisions in ways that deviated from their personal opinions. Veterinary officers all agreed that the absence of suffering, pain, harm, or severe fear as legally relevant categories for immediate action did not automatically mean that the horse’s QoL was good.

### Challenges regarding QoL assessments in the context of veterinary decisions and ways to deal with them

Overall, the challenges the non-veterinary participants discussed tended to be about knowing what was best for the horse based on the horse’s QoL, while the challenges veterinary professionals discussed tended to be related to other stakeholders in the process of finding out and doing what was best for the horse.

#### Challenges related to the horse

Various issues were described as challenging when assessing a horse’s QoL or making decisions about veterinary interventions. Decisions about euthanasia accounted for a large share of the challenges. These decisions often came down to the question of “How long is [life] worth living for the horse?” (FG7-own6). The fundamental challenge with finding the right answer was, in the words of a horse owner, “that the horse can’t talk to us, [can’t tell us] what it would like.” (FG7-own2). A farrier stated that making the right decision was difficult because “[…] at the end of the day, these are all decisions, and whether they were right or wrong is something that simply doesn’t become clear until later.” (FG3-far4).

Participants in both veterinary and non-veterinary groups described the decision about when to euthanise a horse as particularly difficult in two contexts. Firstly, if the horse’s condition had been slowly deteriorating over an extended period with no expectation of improvement and, secondly, if the horse’s condition had been or still was going up and down. For example, a caregiver said that it was difficult if a veterinarian had been treating a severely unwell horse for several days “[…] and there has been no improvement through several days and then the decision really is, do you put it out of its misery now or do you really wait again [to see] whether it will really get better again in six or five days.” (FG4-care4).

Horse owners, veterinary professionals, and one farrier described decisions about euthanasia as particularly difficult when different aspects of a horse’s life, such as the horse’s health and the horse’s behaviour, resulted in conflicting evaluations of the horse’s overall QoL. One horse owner described a decision about a friend’s horse as difficult when considering the horse’s behaviour, “[…] because actually from his prognosis, from his state of health, that’s actually not good. But if I only look at the horse and don’t know that he got such a bad diagnosis, then I find it difficult.” (FG6-own3).

#### Disagreements among stakeholders

Participants, especially in the horse owner groups, mentioned that there were different opinions about what was good for a horse, not only “in the horse sector” (FG6-own1) in general but also among veterinarians. A horse owner stated that *“*[…] for me, [just] the decision about which veterinarian I call is difficult. If you ask three different veterinarians about a more serious problem, then you get five different opinions [laughs], and then you still have to figure out for yourself who you should believe now, exactly.” (FG6-own4).

In contrast, a veterinary officer said that there were “[…] sometimes very dogmatic attitudes […]” (FG2-offvet3) but “[…] not among veterinarians but among horse people […]” (FG2-offvet3). The veterinary professionals in the study only rarely mentioned disagreements among veterinarians (e.g., about how long horses should be treated to advance novel approaches in a clinical setting). Instead, they described disagreements with horse owners as a major challenge when assessing QoL and in relation to euthanasia decisions. An equine veterinarian explained, “When I get somewhere as a veterinarian, it’s mostly different from what the owner describes. The owner always believes that everything is OK. It often diverges a lot.” (FG1-vet4). Veterinary officers echoed this view. They acknowledged that horse owners might have the best of intentions but reported, in the words of one of them, that horse owners often had an “[…] idealised representation of reality.” (FG2-offvet5).

Equine veterinarians described convincing horse owners of the severity of their horse’s condition as a major challenge in cases where the owners disagreed with their advice. An equine veterinarian said, *“*I believe that is the worst thing, when you can’t influence the owner enough.” (FG1-vet3). The data allowed us to identify horse owners’ emotions as an underlying challenge in the context of such disagreements. For instance, one equine veterinarian reported: “[…] I can say what I want now. She was crying and said, ‘I can’t lose another horse now.’ And I said: ‘At this moment, it’s not about you but about your horse’ […]” (FG1-vet3). Horse owners talked about the emotional component of euthanasia decisions, which one of them described as “[…] emotionally simply the hardest decision.” (FG7-own1). Many owners also acknowledged that being emotionally close to a horse could hinder an accurate assessment of the horse’s QoL, and some voiced criticisms of other owners they had encountered. Some horse owners also acknowledged their own limitations; one of them said, “[…] I think—I don’t exclude myself from that—that one can’t always really judge it 100% alone because of the emotional component.” (FG5-own5).

#### Strategies for dealing with challenges regarding QoL assessment and decision-making

Participants described various strategies for dealing with challenges related to assessing QoL and making decisions about euthanasia. We identified exchanging opinions with other people as a relevant strategy for dealing with challenging decisions. Horse owners and caregivers in particular mentioned talking to a veterinarian, to colleagues, or to friends and family to gather opinions or to make decisions together. A horse caregiver in a sanctuary context, for example, said that they discussed decisions as a team and noted that “If everyone then decides the same thing, that, of course, helps.” (FG4-care5). However, opinions about a horse could differ and when exchange with others did not bring clarity, relying on one’s personal evaluation of a horse was identified as a strategy for dealing with that. It was, one horse owner said, “[…] very important that you make the right decision for yourself” (FG6-own1). An equine veterinarian echoed that view: “[…] for me, decision-making is also first of all about being at peace with myself what I think about a case.” (FG1-vet4).

Another strategy participants in all groups used was drawing parallels between the horse’s situation and human experiences to address uncertainty about the horse’s perspective on the situation. A horse owner said, “[…] I often think to myself, what would it be like if it were my grandmother? Would my grandmother, because she has aches now and then, no longer want to live? Or would my grandmother, because she cannot eat well, no longer want to live? Or would she perhaps indeed want to? I act like that a little bit, I must say.” (FG5-own3). Equine veterinarians gave examples of using comparisons to human experiences to convince horse owners of the need for action. One equine veterinarian said, “The more drastic, the more blatant the comparisons can be and the more the owners can live that and imagine it, the easier you get through [to them], that it really comes to a change [in their view].” (FG1-vet5). However, participants in both veterinary and non-veterinary groups also criticised projections of human needs onto horses. One horse owner, for example, said that it was important to realise that “[…] the horse has needs that have nothing to do with the needs of a human.” (FG7-own6).

One equine veterinarian’s strategy for handling disagreements with horse owners was to encourage horse owners to keep a “diary” (FG1-vet6) to track the number of good and bad days for their horse. Equine veterinarians also mentioned the option of involving a veterinary officer as a last resort to enforce a decision for a horse if all other approaches failed.

## Discussion

The study provides insights into how equine stakeholders, including horse owners, equine veterinarians, veterinary officers, farriers, and horse caregivers, use and assess equine QoL, particularly in the context of making decisions for chronically ill or old horses. Our unique framework of including the various stakeholders in one study that focused on the QoL of old or chronically ill horses enabled us to identify similarities and differences between the stakeholders involved in care and decision-making. Our findings show that there was agreement about the importance of the individuality of the horse for QoL assessments and the importance of QoL in making decisions about veterinary interventions. However, results show that differences occur between the groups with respect to what is required to assess QoL, the weight the contributions of different stakeholders to QoL assessments should have, and the challenges they experience regarding assessments of equine QoL and decision-making. In addition, the study sheds light on the strategies stakeholders employ to deal with the various challenges they face during QoL assessments and decisions about veterinary interventions.

### The role of QoL in decision making and important aspects for QoL assessment

Across the groups, the various stakeholders agreed that the individuality of the horse was central for assessing a horse’s QoL. This was based on the understanding that horses have distinct characteristics that necessitate an individualised approach to their husbandry and care. This emphasis on the individual horse and the horse’s needs is in line with findings of other studies [24, 44]. For instance, stakeholders from the British horse-racing industry reported consideration of the individual characteristics of a horse as important if a horse is to live the ‘best life’ as opposed to following a ‘one-size-fits-all’ approach in order to fulfil minimum welfare requirements [44].

Our findings show that the horse’s current and predicted QoL during and after treatment are of particular importance in the decisions various stakeholders make. These findings confirmed our expectations and are in line with other studies showing that horse owners consider QoL as an important factor for decisions about euthanasia and care for their horses in general [3, 5, 7–10].

However, we identified disagreement among stakeholders with respect to the time needed to assess a horse’s QoL. Closely related to this, veterinarians, including veterinary officers, and horse owners had conflicting opinions about the importance of their own and others’ contributions to QoL assessments. Non-veterinary participants emphasised that QoL assessments should ideally be done over longer periods. This is in line with UK leisure horse owners’ descriptions of monitoring their horse’s well-being as a constant process over time [24]. Correspondingly, horse owners emphasised their own importance for assessing their horse’s QoL because they know their horse well. However, horse owners also voiced criticism of other owners or themselves regarding how they assess equine QoL. We know from other studies that horse owners find their own contributions to assessments of well-being important but sometimes criticise how other horse owners assess well-being [24, 25]. The veterinarians in our study focused on criticising horse owners’ QoL assessments and did not acknowledge their contributions as valuable. This was not what we expected and contrasts with how QoL assessments are discussed in the literature, which often highlights the relevance of the owners’ perspective [4, 21, 22]. There were some instances in our study of veterinarians describing owners in positive terms for putting a lot of effort into the care and management of their horses. However, in general, the equine veterinarians and veterinary officers in our study presented horse owners’ contributions to QoL assessments rather negatively and described their interactions with owners regarding equine QoL as challenging. The equine veterinarians in our study did not discuss time constraints as a problem in the context of QoL assessments, although they acknowledged that seeing a horse in different contexts and at different times can provide valuable additional information. This is an interesting finding because it not only highlights a difference between what different stakeholders present as important for QoL assessment but also because not having enough time per patient has been identified as a contributing factor to workplace stress for small animal veterinarians [58].

That the equine veterinarians in this study did not discuss a lack of time as a crucial issue or recognise the owner’s “social capital” [24] derived from the owner’s relationship with their own horse may be explained by four things. First, the veterinary professionals highlighted their expertise and experience with horses. In combination with their outsider’s perspective on a horse, their training and experience probably enables them to identify issues quickly. Second, the fact that we asked participants about difficult decisions made it likely that they would focus on negative experiences. However, the fact that the members of both veterinary groups began discussing issues with horse owners early in their sessions seems to indicate that they experience a high level of frustration in their daily practice. This is not surprising, as conflicts with clients are a known stressor for veterinarians in general [59–61]. However, that horse owners misjudge both the health of their horses and behavioural signs of distress [33–37] does not mean that veterinarians are always correct or in agreement about their assessments. Veterinarians sometimes disagree with each other, a fact that they find challenging [59]. For instance, Sellon et al. [62] found that assessments of pain levels varied widely among horse owners but also among veterinarians. Third, veterinarians in our study based their contributions during the focus group discussions on cases they had experienced that may have been on the extreme end of the scale. The perceived inadequacy of the horse owners in these scenarios may have outweighed any potential insecurities veterinarians and veterinary officers felt about the horse’s QoL possibly resulting from a short consultation time. Fourth, the veterinarians’ disagreements with owners about the relevance of owners’ perspectives in assessing QoL may be due to an underlying difference in how stakeholders conceptualise QoL. Understanding QoL as mainly related to physical health would make it straightforward and relatively fast for veterinarians to assess a horse’s QoL. We did not find direct evidence of this in our data, however, since all stakeholders highlighted the importance of a wide range of aspects. Nonetheless, horse owners indicated that their contributions focused on the mental and emotional aspects of QoL which typically required a longer observation period and a recognition of differences over time.

### A collaborative approach to QoL assessment

Keeping in mind that stakeholders bring different perspectives onto a horse’s QoL, stakeholders would ideally follow a collaborative approach to QoL assessments that explicitly considers a variety of issues that are relevant for a horse’s QoL. Each of those issues can be seen as one piece of a mosaic. Assembling all the pieces of the mosaic creates a unified whole, representing the horse’s QoL. By explicitly discussing the different aspects that belong to a horse’s QoL and integrating them into a whole in a collaborative process, our findings indicate that this approach would arrive at a richer QoL assessment. In particular, non-veterinarians pointed out the importance of veterinary input when determining their horse’s condition and making decisions about veterinary care. This corresponds with the findings of previous studies [3, 5, 6, 8, 26, 27, 38] and suggests that relevant persons in a position to assess the QoL of an individual horse follow a collaborative mosaic construction approach at least some of the time.

However, findings of our study show that stakeholders switch between a holistic approach, i.e. intuitively rather than explicitly combining relevant information, and a collaborative mosaic assembly approach. Participants were vocal about not being able to reduce QoL assessments to a limited set of questions or facts, Instead, they emphasised the importance of seeing the horse and considering a large number of aspects of the horse’s life to get an overall impression. In combination with referring to whole-animal measures to capture the horse’s overall state from the horse’s perspective [63], such as the horse’s will to live, the stakeholders therefore appeared to consider their own assessment as holistic. In practice however, it seems unlikely that such an intuitive approach is ideal, as it frequently results in disagreements. As Smith et al. [24] point out, many aspects of the human-horse relationship colour how a person interprets a horse’s well-being. When a stakeholder uses a holistic approach that relies on intuition and unconscious integration of different aspects, their ability to explain a QoL assessment is limited. It is hard to convince someone that a “feeling” about the horse’s state is correct when the other person has a different “feeling” about it. The frequent disagreements our participants described are a testament to this.

### The potential for QoL assessment tools

QoL assessment tools can provide a list of criteria relevant to QoL for users to go through and combine into an overall evaluation, thereby fostering a collaborative mosaic assembly approach. This can reduce the danger of applying a reductionist piece-of-a-mosaic approach to QoL assessment; that is, knowingly or unknowingly focusing only on one aspect of QoL. However, participants in our study did not discuss using formal assessment tools. As we did not specifically ask about the use of QoL (or welfare) assessment protocols, this finding is not surprising. It is also in line with other studies that show that horse stakeholders do not use formal assessment tools and only rarely keep logs of the state of their horses [20, 24]. Possible reasons for this are the practicability of and effort required to use formal protocols, the lack of a perceived need to receive support for well-being assessments [24], and the lack of an adequate protocol for QoL assessments for chronically ill or old horses [42]. Our findings suggest that any QoL assessment tool that aims to appeal to stakeholders must take the importance of the horse’s individuality into account. This implies, for example, that the parameters in an assessment protocol must be weighed in a way that facilitates a contextualisation of the assessment to the individual horse.

QoL assessment tools face the challenge of balancing sensitivity to a horse’s individuality with providing useful guidance through formalisation of the process. Improving horse-based indicators of equine QoL could help reduce some of the uncertainty regarding the state of a horse. However, a QoL assessment tool cannot solve the ethical question of which level of QoL is acceptable for a horse in a particular situation. In addition to that, QoL assessment does not consider aspects beyond the horse’s QoL that can influence a decision about veterinary care. A QoL assessment tool should therefore not be confused with tools designed to support decision-making that is focused on the ethically challenging aspects of a decision situation such as the Veterinary Ethics Tool [64] and targeted approaches to supporting end-of-life decisions in veterinary contexts [65–67].

### Strategies for dealing with challenges resulting from QoL assessments and decision-making

Rather than resorting to QoL assessment protocols, the participants in our study discussed other strategies for dealing with challenges resulting from QoL assessments and decision-making. There were common threads among the groups but also differences because the groups focused on different challenges. Anthropomorphising a horse’s situation was a key strategy for stakeholders in the context of assessing QoL and making decisions about veterinary care. It fulfilled two different functions: as a basis for making a decision and as a means of convincing other stakeholders to make a particular decision. Particularly in the non-veterinary groups, participants drew parallels and made comparisons between humans and horses when they discussed making a decision or justifying a course of action. This is not surprising, because the concept of QoL was initially developed for humans [68] from a human perspective.

Non-veterinary participants used their perceptions of a horse’s will to live as a point of reference that served to elucidate and bolster decisions that would otherwise be characterised by uncertainties. During discussions of personal experiences, the participants also invoked their perceptions of a horse’s will to live as a justification for decisions they had made in the past. Some scepticism is warranted about the existence of such a will to live (or to die) for horses. Although some argue that animals can have a concept of death, that does not inevitably translate into an awareness of their own mortality [69, 70]. Thus, the horse’s behaviour might be more reflective of their overall energy level at the time rather than an expression of a wish to live or die.

In the veterinary groups, participants referred to comparing a horse’s condition to human experiences as a way to convince other stakeholders that the veterinarian’s assessment was correct. These findings show how an intentional use of comparing human and animal experience, such as painful illnesses, can benefit the animal. Such comparisons can bridge the gap between the owner’s knowledge and understanding and the knowledge and understanding of the veterinarian. In addition to that, using human experiences as a reference point to relate to and compare with can also support mutual understanding between stakeholders in general. This is not limited to veterinarians convincing horse owners but can be used between other stakeholders in general to convey their interpretation of the horse’s situation to each other. Anthropomorphising an animal’s situation or condition can have negative effects for animals in human care [71] if the animals’ needs are missed or misunderstood. However, our findings show that it can also fulfil important functions that can benefit the animal.

Another important strategy described mostly in the non-veterinary groups was discussing a challenging decision with other people. Horse owners are known to seek advice from both equine professionals and non-professionals [3, 5–9, 26, 27, 38]. When another person’s assessment agrees with that of the owner, it can be a relief for the decision-maker. But it is not always the case that another person will agree with an owner’s assessment, and participants in our study also described how they had to be at peace with their own judgements first and foremost. Widening horse owners’ frames of references to include new learnings and perspectives on their horse is one way to encourage horse owners to consider new ideas [24]. That horse owners and caregivers describe seeking exchange with others is a positive step in this direction. However, if that advice-seeking or discussion is limited to attaining social validation for beliefs an owner already has [24], the chance that the owner will acquire new perspectives is small and exchange with others can lead to a false sense of being right about a decision. Facilitating communication and reflection among different stakeholders is therefore important.

Ideally, conversations about QoL between veterinarians and owners would be part of routine veterinary examinations so that a shared understanding of the concept and what it means for an individual horse can be developed and discussed over time. Rollin suggests devising a list of “what makes the animal happy or unhappy, and how he or she [the owner] knows” [72]. This would also encourage owners to prepare for challenging decision situations such as when to euthanise a horse. In addition, short checklists at the beginning of consultations can address ethical questions related to a case such as “Would treatment really be in the animal’s interest? What is the expected end state for this animal? What does the animal have to go through on the way?” [73]. A recent study on interactions between veterinarians and horse owners highlighted how important it was that horse owners’ values, their reasons for seeking treatment for their horse and the way they construct knowledge of their own horse were recognised by veterinarians for owners to adopt a veterinarian’s advice [74]. There are some similarities to the context of paediatric care where parents and health care providers strive for shared decision-making and patient preferences are mainly communicated through the patient’s parents. Parents have been found to value clarity about the time horizon of a decision, the recognition of patient preferences and recognition of inherent uncertainty in decision-making as positive for shared decision-making between them and the health care providers [75]. These aspects are likely also beneficial to consider in the context of veterinary interventions.

### Limitations of the study

There are some limitations to the present study. Only those stakeholders with a particular interest in making good decisions for their old or chronically ill horses will have been motivated to take part in the study. The participants are not necessarily representative of all stakeholders. However, that was not a problem for our study, as we were interested in how stakeholders struggle with QoL assessments and decision-making when they are invested in making good decisions for a horse. Another potential limitation is the fact that participants with strong opinions can dominate the discussion and the overall opinion in a group discussion [76]. The moderator of the group discussions for this study tried to soften this dynamic by steering the conversation when necessary and making sure that all participants got the chance to contribute. Another limitation is that in a focus group study, the presence of the moderator shapes the discussion and influences participants [77]. Although this cannot be fully avoided, we increased the consistency of the interviewer’s influence between groups by using a structured interview guide and by refraining from expressing personal opinions during the discussions. Participants have been categorised as owners, caregivers, farriers, veterinarians, or veterinary officers, even though some of the participants fit more than one category because they were equine professionals but also horse owners. We focused on their professional role related to horses because we expected that role to have the greatest impact on their answers to the questions asked in the focus group study. Future studies could include further stakeholders such as physiotherapists and other professionals who provide relevant care for chronically ill or old horses. In addition, future studies could seek to include more male horse owners. Finally, the findings were generated for Austria and may have limited relevance for other countries. Austrian animal welfare law permits the killing of animals only for a reasonable cause and requires animal owners to initiate veterinary care if it is required (Austrian Animal Welfare Act 2004, § 6 [78]). This was not directly discussed by the participants but is likely to influence decision-making in practice. This makes our findings particularly relevant for countries with similar laws.

## Conclusion

Based on our findings, we conclude that QoL is an important concept in decisions about veterinary care for old or chronically ill horses. Furthermore, the various stakeholders agreed about the importance of focusing on the individual horse and the suitability of the horse’s living conditions for this individual when assessing the horse’s QoL. Horse owners and caregivers highlighted their knowledge of a horse and the relevance of the time they spend with their horse. In contrast, the veterinarians focused on the differences between their QoL assessments and those of horse owners whose assessment veterinarians criticised as often too positive.

We identified different challenges related to assessing QoL and making decisions about veterinary care. Non-veterinarians faced difficulties with assessing QoL and making the right decisions, whereas veterinary professionals’ challenges focused on convincing horse owners that their veterinary assessments were correct. The study identified a variety of strategies for addressing these challenges. For non-veterinary participants, talking to other people to gain new information and perspectives can be helpful. For both non-veterinary and veterinary participants, comparing a horse’s condition with human experiences of ageing and illness was a common strategy for overcoming challenges with assessing equine QoL and for making decisions about equine care.

### Electronic supplementary material

Below is the link to the electronic supplementary material.

Additional File 1: Details of the recruitment process: Details of the recruitment process for the different focus groups with number of focus group, recruitment channel and number of recruited participants for the different recruitment channels.

Additional File 2: Interview guide for focus group discussions on ‘Decisions for chronically ill and old horses’. Interview guide used for the focus group discussions of horse owners (groups 5–7) with adjustments to the interview guide for the other groups indicated in the text.

Additional File 3: Reflexivity statement. Reflexivity statement and professional background of project team members.

Additional File 4: Code list for focus group study ‘Decisions for chronically ill and old horses’. Final categories and codes used for all focus groups with abbreviations and descriptions.

## Data Availability

The datasets used and/or analysed during the current study are available from the corresponding author upon reasonable request.
