# Supplementary material for: “How long is life worth living for the horse?” A focus group study on how Austrian equine stakeholders assess quality of life for chronically ill or old horses
Source: BMC Vet Res. 2024 Aug 6;20:347. doi: 10.1186/s12917-024-04211-8 (PMC11302025; doi:10.1186/s12917-024-04211-8)
Supplement: Supplementary file 1 — Additional File 1: Details of the recruitment process: Details of the recruitment process for the different focus groups with number of focus group, recruitment channel and number of recruited participants for the different recruitment channels. [file 12917_2024_4211_MOESM1_ESM.pdf]

Additional File 1

Details of the recruitment process

Additional Table 1: Details of the recruitment process for the different focus groups with number of focus group, recruitment channel and number of recruited participants

<table>
<tr><th>Nr.</th><th>Group</th><th colspan="2">Number of recruited participants</th></tr>
<tr><td rowspan="6">1</td><td colspan="3">Equine veterinarians</td></tr>
<tr><td>Facebook post by the University of Veterinary Medicine, Vienna
▪ on 3rd June 2021</td><td rowspan="2" colspan="2">5 veterinarians</td></tr>
<tr><td>Non-personalised email invitation to all members of the Austrian association of equine veterinarians (“Vereinigung Österreichischer Pferdetierärzte”)
▪ on 7th June 2021
▪ 316 members (as of 20th April 2023)</td></tr>
<tr><td>Email invitations to 25 equine veterinarians
▪ systematic random sampling stratified according to Austrian federal states
▪ based on the publicly available online directory of veterinarians who are members of the Austrian association of equine veterinarians (“Vereinigung Österreichischer Pferdetierärzte”)
▪ on 27th May 2021</td><td colspan="2">1 veterinarian</td></tr>
<tr><td>Personal contacts of the research team / snowballing</td><td colspan="2">1 veterinarian</td></tr>
<tr><td>Brief presentation of study and invitation at Alumni evening for equine veterinarians
▪ on 11th June 2021</td><td colspan="2">---</td></tr>
<tr><td rowspan="4">2</td><td colspan="3">Veterinary officers</td></tr>
<tr><td>Facebook post by the University of Veterinary Medicine, Vienna
▪ on 3rd June 2021</td><td></td><td rowspan="3">1 veterinary officer</td></tr>
<tr><td>Non-personalised email invitation to all members of the Austrian association of veterinary officers (“Österreichischer Verband der Amtstierärztinnnen und Amtstierärzte”)
▪ on 5th May 2021
▪ 210 members (as of 20th April 2023)</td><td></td></tr>
<tr><td>Email invitation to all heads of the Austrian federal veterinary offices (“Landesveterinärdirektoren”) with a request to distribute the study to the local veterinary officers
▪ on 26th May 2021</td><td>6 veterinary officers*</td></tr>
<tr><td rowspan="2">3</td><td colspan="3">Farriers</td></tr>
<tr><td>Facebook post by the University of Veterinary Medicine, Vienna</td><td colspan="2">2 farriers</td></tr>
</table>

	▪ on 3rd June 2021	
	Non-personalised email invitation to all members of the Austrian association of farriers ("Österreichischer Hufschmiedverband")<br>▪ on 22nd September 2021<br>▪ 108 members (as of 20th April 2023)	
	Personal contacts	2 farriers
**4**	**Horse caregivers**	
	Facebook post by the University of Veterinary Medicine, Vienna<br>▪ on 3rd June 2021	1 horse caregiver
	Non-personalised email invitation to all alumni via email list of school for animal caregivers at the Vetmeduni ("Tierpflegeschule Vetmeduni")<br>▪ on 27th July 2021 and 14th September 2021	---
	Email invitation to three equine hospitals / horse farms on 17th September 2021 and on 6th October 2021	---
	Personal contacts of the research team / snowballing	4 horse caregivers
**5-7**	**Owners of horses for income and for leisure use**	
	Facebook post by the University of Veterinary Medicine, Vienna<br>▪ on 3rd June 2021	15 horse owners
	Personal contacts of the research team / snowballing	1 horse owner
**7**	**Horse owners from a sanctuary context**	
	Facebook post by the University of Veterinary Medicine, Vienna<br>▪ on 3rd June 2021	
	Direct invitation via email to 11 shelters/sanctuaries<br>▪ on 28th May 2021, reminder email on 13th July 2021<br>▪ animal shelters and sanctuaries found through trade directory "herold.at" (shelters) and Google search (sanctuaries)<br>▪ inclusion criteria: keeping horses and being registered as an animal shelter or animal protection society	1 horse owner

* One of these veterinary officers dropped out at the beginning of the discussion for technical reasons
