# Supplementary material for: “How long is life worth living for the horse?” A focus group study on how Austrian equine stakeholders assess quality of life for chronically ill or old horses
Source: BMC Vet Res. 2024 Aug 6;20:347. doi: 10.1186/s12917-024-04211-8 (PMC11302025; doi:10.1186/s12917-024-04211-8)
Supplement: Supplementary file 2 — Additional File 2: Interview guide for focus group discussions on ‘Decisions for chronically ill and old horses’. Interview guide used for the focus group discussions of horse owners (groups 5–7) with adjustments to the interview guide for the other groups indicated in the text. [file 12917_2024_4211_MOESM2_ESM.pdf]

Additional File 2

Interview guide for focus group discussions on ‘Decisions for chronically ill and old horses’

The overall aim of the focus group study is to gain insights into how horse owners, equine veterinarians, veterinary officers, farriers, and horse caregivers assess and use equine QoL. The following interview guide was used for the focus group discussions of horse owners (groups 5-7). Adjustments to the interview guide for the other groups are indicated in the text. The most important adjustments were made concerning the introductory questions and the question about decisions related to veterinary care, which were perceived as challenging. The latter was modified to refer to the professional context for equine veterinarians, veterinary officers, farriers, and horse caregivers.

Overview:

Total time: 2.5 hours including 15 min break (150 min in total).

**Log in/ set up**	**(5 min)**
**Welcome and introduction by moderator**	**(5 min)**
**Round of introductions**	**(20 min)**
**Quality of life of horses (via fictional example horse *Dusty*)**	**(51 min)**
**BREAK**	(15 min)
**Decisions about veterinary interventions**	**(20 min)**
Via the participants' experiences	
**Case examples**	**(30 min)**
	(Total: 146 min + 5 min buffer)

Aspect	Text	Overview
**Welcome and introduction**	**Step 1: Welcome and introduction: 10 min (5 min talking, 5 min for technical issues)**<br>__A:__*(Please say everything!)*<br><br>Welcome to this virtual space of the Messerli Research Institute in Vienna.<br>Thank you for participating in this focus group study on "Decisions for chronically ill and old horses." We look forward to the next 2.5 hours. We will have a 15 min break about halfway through.	 **Welcome and introduction**<br><br>Organisational aspects:<br>- Headsets, Sound test<br>- Consent forms and data protection<br>- Recording of the conversation

	Before we get into the topic, I would like to introduce myself briefly:<br>*[HG introduces himself]*<br><br>[ML] is also present to assist with the process. [ML] will take additional notes during the discussion and keep track of the time.<br>I would like to point out right at the beginning and apologise for the fact that I will be moderating this conversation quite tightly and will also interrupt you at one point or another to do so, so that we can cover all aspects of the study. I ask you not to interpret this as rudeness on my part. I just want to make sure that we stay within the agreed time frame and can discuss all the planned topics with you.<br><br>Before we start, a few technical details:<br>▪ You have all received a consent form from us, which you have signed and returned to us. Thank you very much for that. I would like to briefly mention the most important points again.<br>o Since we cannot remember everything that is said and discussed, this **conversation is recorded**. The recordings will be treated confidentially and will only be used by us for research purposes. The **analysis will take** place in an **anonymised form, so** that names will also not appear in scientific publications. We ask you to treat what is said here as **confidential as** well.<br>o In order to understand each other well and keep to ourselves, we all use a **headset**.<br>o Do you have any questions about the **consent form** or data protection?<br><br>▪ If you have any content-related **questions about the project** of which this study is a part, we will be happy to answer them **after the interview**. Please understand that we are keeping the introduction short in order not to influence you too much.<br>Before we start with a short round of introductions, I would like to emphasise that we are **interested in your thoughts, attitudes and opinions**. **Everything that comes to your mind and that seems important to you is important to us.** We are interested in your experiences, which will be used to better understand and analyse the topic of "decisions for chronically ill and old horses". **We thank you for engaging in the process and sharing your thoughts with us**.	

	I will do my best to make sure that you can all have your say. If you think of something, please raise your **hand, virtually or to the camera**. If you go to the emoji icon at the bottom of the bar, you can select "raise your hand" or "lower your hand".<br>If possible, please **avoid speaking at the same time,** as you will then unfortunately not be understood for technical reasons.	
**Introduction**	**Step 2: Introductions: Time limit 20 min**<br><br>**A:** We would like to start with a small **round of introductions** in 2-3 sentences, where you have the opportunity to introduce yourself with your name and some information about when and in what way you have experience with horses.<br><br>***Variations****:*<br>***Horse owners*** (groups 5-7): We would also like to ask you to tell us briefly how old your own horses are and how and for which purpose you keep them.<br><br>**Veterinarians** (group 1): We would also like to ask you to tell us briefly in which context (e.g. employed in a clinic or self-employed) and since when you have been working as a veterinarian for horses.<br><br>**Veterinary officers** (group 2): We would also like to ask you to tell us briefly for how long you have been working as a veterinary officer and how much you encounter horses in your role.<br><br>**Farriers** (group 3): We would also like to ask you to tell us briefly what kind of training you have had in the field of hoof care, where you see your focus in your work and how long you have been working as a farrier.<br><br>**Horse caregiver**s (group 4): We would also like to ask you to tell us briefly how long you have been working as a horse caregiver, in what kind of stable you are employed and	**Round of introductions**<br><br>Participants briefly introduce themselves (4-5 sentences)

	what your tasks are. In addition, we are interested in whether you also work with chronically ill or old horses.<br><br>***Checklist:***<br>○ ***All*** *participants introduce themselves by name and speak 4-5 sentences about themselves.*<br><br>**Click**	
	**Part 1: Quality of life: 51 min total**	
**Part 1**<br>**Quality of life**	<u>**A**</u>: Now I'd like to begin and talk to you about a horse, Dusty. [*Show image]*<br>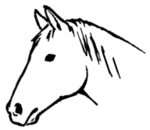<br>**Click**<br><br>**Step 3: Time limit 3** min<br><u>**A:**</u> You don't know Dusty. What would you want to ask me to learn more about Dusty and his life? **Please write down the first three questions that come to your mind.**<br><br>**Click**<br><br>*The participants write down three questions. The facilitator then asks what the participants have written down.*<br><br>**Step 4: Time limit 10 min**<br><br><u>**A:**</u> Who would like to start and name their three questions?	Here we find out which aspects of a horse's life the participants find interesting. This is relevant because it allows us to compare whether and how these aspects are also reflected in the assessment of quality of life.

	The facilitator asks ***all*** *participants:*<br><br>Which questions did you also write down and which ones did you write down in addition? I would like to ask you to expand only briefly on the additional questions.<br><br>***Checklist****:*<br>○ ***All*** *participants name what spontaneously interests them about a horse's life.*	
	**Step 5: Time limit 15 min**<br><br>*After all aspects have been mentioned by the participants, the moderator asks:*<br><br>**A: If you imagine that I answer the questions you have just mentioned, would this information be enough for you to assess how Dusty's quality of life is? Or would you want to know something else?**<br><br>**Click**<br><br>*The participants (probably) name further points. It may be necessary to ask for more details.*<br><br>*The facilitator addresses the other participants:*<br>▪ **Does anyone else have another aspect that he or she finds important when it comes to assessing Dusty's quality of life?**<br>▪ What else would you like to know in order to assess Dusty's quality of life?<br>▪ Did you come up with similar aspects?<br><br>***Checklist****:*<br>○ *It becomes clear whether there is a difference between what people are generally interested in and what is relevant for the assessment of quality of life.*<br><br>**Step 6: Time limit 3 min**<br>**Click**	After the general question above about which aspects are of interest to the participants, here we explicitly ask about the relevant aspects for assessing the quality of life.<br>It would be possible here that the participants either add other aspects, emphasise aspects or also say that some points are not relevant.<br><br>Furthermore, the aspects are to be weighted here.

	**A: Please now briefly write down a ranking of the three most important pieces of information.** What is the most important, second most important and third most important information for assessing Dusty's quality of life?<br><br>*Participants write down a list of the most important information.*<br><br>**Click**<br>**Step 7: Time limit 20 min**<br><br>*The facilitator asks for the information according to importance and enters the discussion.*<br><br>A: What is ranked as the most important information for you?<br><br>Has anyone here written anything else down or would like to comment on the aspect mentioned?<br><br>What is the second most important piece of information for assessing Dusty's quality of life?<br><br>What is the third most important piece of information for assessing Dusty's quality of life?<br><br>Questions for discussion:<br>▪ Why is this information particularly important for assessing Dusty's quality of life?<br>▪ How do you experience situations in everyday life *[Veterinary officers: in practice]* with horses where you lack this information?<br>▪ How does this information help you *[Veterinary officers*: *in practice*] to assess the quality of life of horses?<br>▪ Can you illustrate your point with a concrete situation?<br>▪ Can you tell me about a situation where you were unsure of what was most important for the assessment of QoL?<br><br>***Checklist:***<br>o *Particularly important aspects for the assessment of quality of life have become clear.*	

	○ *Do the participants agree or disagree on the weighting?*<br>○ *Reasons for the importance of the individual aspects for the assessment of quality of life were given.*	
	**Step 8: Break: Time limit 15 min**<br><br>*Maybe:* In the course of our conversation, veterinary treatments have also come up as a topic. We will take this up after a short break.<br><br>**A:** We will now take a break for 15 minutes. It is best to stay logged into the meeting and just turn off the camera and microphone.<br><br>**Click**<br><br>**A:** We will be back at *TIME.*<br><br>*Note the time on the slide for when the discussion continues.*	
**PAUSE (15 min)**		
	**Part 2 Decisions on veterinary interventions**	
**Part 2 Decisions about therapies and treatments**	**Step 9: Time limit 20 min**<br><br>**A:** In the next part of the interview, we would like to look at decisions about veterinary treatments.<br><br>***Variations***:<br><br>**Horse owners** (groups 5-7): Here we are interested in your experiences. **Can anyone tell me about a situation in which you found it difficult to make a decision about veterinary treatment for your chronically ill or old horse?**	Experiences with medical treatment decisions<br><br>The question about the problems makes it clear which aspects play a role for the horse owners when making decisions.<br>The question of what was ultimately decisive or what would have changed the decision is used to reveal the weight of the decision-making factors.

Veterinarians (group 1): Here we are interested in your experiences as an equine veterinarian. Can anyone tell me about a situation in which you found it difficult as a veterinarian to make a decision about veterinary treatment for a chronically ill or old horse?
Here it should be clear that this is about horse patients and not about their own horses.

Veterinary officers (group 2): Here we are interested in your experiences. Can anyone tell me about a situation in which you found it difficult as a veterinary officer to make a decision about veterinary treatment for a chronically ill or old horse?
Here it should be clear that this is about horse patients and not about their own horses.

Farriers (group 3): Here we are interested in your experiences as a farrier. Can anyone tell me about a situation in which you found it difficult as a farrier to appraise a decision about veterinary treatment for a chronically ill or old horse?
This is about horses that are looked after as farriers (not their own).

Horse caregivers (group 4): Here we are interested in your experiences as a horse caregiver. Can anyone tell me about a situation in which you found it difficult as a horse caregiver to appraise a decision about veterinary treatment for a chronically ill or old horse?
This is about horses that are looked after as horse caregivers (not their own).

[***Variations*****:** *In the following questions 'decisions' was replaced with 'appraisals' and 'to make a decision' with 'to appraise a decision' for farriers and horse caregivers. A question was added for all groups.*]

- What were the problems in this situation?
- Why was the decision [*appraisal*] difficult in this situation?
- What would you have needed to make [*appraise*] a decision more easily?
- What information did you miss to make the decision [*appraisal*] easier?
- What was ultimately decisive for your decision [*appraisal*]?
- What would have made you decide differently in that situation [appraise the situation differently]?

	▪ In retrospect, do you still think the decision was right?<br>▪ [*In retrospect, do you still think the decision was wrong?*]<br><br>*The facilitator invites the other participants into the discussion:*<br><br>▪ Do you share these experiences?<br>▪ Do you find decisions [*appraisals*] in such situations similarly difficult?<br>▪ Do you also find such situations difficult?<br>▪ Do you find such decisions [*appraisals*] easy, or do you also experience them as difficult?<br>▪ What brings you certainty for your decisions [*appraisals*] in such situations that you experience as difficult?<br>▪ What were similar situations where you found it difficult to make [*to appraise*] decisions about the treatment of your / a horse?<br>▪ In case participants do not tell enough: What has made the decision [*appraisal*] easier in other situations?<br><br>*(Please also see the next page).*<br>*Only if it is not mentioned anyway, the moderator asks:*<br><br>▪ What role did the horse's **quality of life** play in the [*appraisal of the*] decision?<br>▪ What role did **exchange with the vet** play in the decision?<br>*Variations:*<br>▪ **Equine veterinarians / veterinary officers:** What role did the exchange with the horse owner play in the decision?<br>▪ **Farriers / horse caregivers**: What role did the exchange with the horse owner or other people play?<br><br>***Checklist:***<br>○ *Situations were described where decisions about medical treatments for chronically ill or old horses were difficult [from the perspective of the respective stakeholder group].*<br>○ *Reasons were given why decisions [appraisals of decisions] were difficult*<br>○ *Aspects that make decisions easy (or easier) were mentioned*	

	○ *It became clear whether the participants had similar or very different experiences.*<br><br>**Click**	
	**Part 3: Case examples: Time limit 30 min in total**	
**Part 3 Case vignettes**	We would now like to look at two fictitious case examples with you. We are interested in how you assess these situations.<br><br>*The facilitator reads the case examples for the participants.*<br><br>**Step 10: Case 1 Time limit 15 min**<br><br>**Case 1:** Due to a recurring eye infection, the gelding Sunny, 24 years old, now also had his second eye removed two years after the removal of his first eye.<br><br>**How do you think this affects the horse's quality of life?**<br><br>**Click**<br><br>**Step 11: Case 2 Time limit 15 min**<br><br>**Case 2:** The vet diagnosed Fly, a 29-year-old Haflinger mare, as 120 kg overweight and with EMS (Equine Metabolic Syndrome). She sets up a feeding and exercise plan that includes increasing exercise and limiting feeding. An essential component of this is limiting grazing time to two hours per day with a grazing muzzle.	The case vignettes are intended to motivate the participants to discuss relevant aspects for the assessment of quality of life and for therapy decisions.<br>The fictional vignettes allow for a discussion without a possibly inhibiting personal reference.<br><br>**Case 1:**<br>▪ General aspects of importance for the constitution and assessment of quality of life<br>▪ Possibly thematisation of age<br>▪ Possibly thematise the housing conditions<br>▪ Possibly addressing the issue of getting used to change<br><br>**Case 2:**<br>▪ Actively limiting the horse's quality of life for improvement/preservation in the future.<br>▪ Weighting of different aspects of importance for quality of life

	The owner has doubts about the measures and says: "But Fly loves to go out to pasture. And what kind of quality of life is that when she is hardly allowed to graze and I am not even allowed to give her a treat?"<br><br>Can you understand the owner's doubts?<br>What would you say to the owner?<br><br>**Additional question:** Would anything be different if Fly was 15 years old and not 29?	▪ Possibly thematisation of the other housing conditions (single box, paddock, other horses etc.)<br><br>***Additional information (if asked):***<br>- 1 % of body weight in hay nets per day<br>- Otherwise stands on sand paddock
**Closing**	**Click**<br><br>**Step 12: Conclusion and thank you: Time limit 1-2 min**<br><br>Finally, we would like to thank you for your time, effort and cooperation. It was a very fruitful and valuable discussion with you.<br><br>As stated in the information letter, you will receive the announced expense reimbursement from us in the next step and we will be happy to send you the published publication if you wish.<br><br>**A: That concludes the official part of the discussion.**<br><br>We will now stay online for a while and will be happy to talk to you if you have any questions or concerns. Otherwise, we would like to thank you once again and wish you a nice day!	Closing
