# Supplementary material for: “How long is life worth living for the horse?” A focus group study on how Austrian equine stakeholders assess quality of life for chronically ill or old horses
Source: BMC Vet Res. 2024 Aug 6;20:347. doi: 10.1186/s12917-024-04211-8 (PMC11302025; doi:10.1186/s12917-024-04211-8)
Supplement: Supplementary file 3 — Additional File 3: Reflexivity statement. Reflexivity statement and professional background of project team members. [file 12917_2024_4211_MOESM3_ESM.pdf]

Reflexivity statement

We acknowledge the active role of the researchers in the production of knowledge.

Our research team consisted of five people with different backgrounds. HG is a moral philosopher and ethicist with additional experience in agriculture. FJ and JMC are equine veterinarians with extensive experience regarding veterinary care for horses and an interest in researching and addressing ethically challenging decisions in equine medicine. SS is a trained non-practising veterinarian who is specialised in empirical veterinary ethics using quantitative and qualitative methods. Her experience with horses in the context of veterinary medicine and personal experiences provide the background for her participation in this research.

ML has a background in animal welfare science and veterinary ethics. Her research focus lies on researching and supporting ethical decision-making in clinical multi-stakeholder contexts in small animal medicine and equine medicine. She is also a trained mediator and has participated in discussions of ethically challenging cases in clinical ethics rounds. Her focus in decision-making situations is on mutual understanding and inclusivity of different perspectives. ML has never owned a horse herself and is not a veterinarian nor a farrier, which supported a more impartial outsider's perspective on the issues studied. However, personal experiences with challenging decisions related to cats' and dogs' veterinary care have shaped and formed her interest in the study subject.

Her role as a researcher and the influence of her experiences and interests on the research process were constantly reflected upon by ML and discussed within the project team as they became relevant to the study. The diverse backgrounds of the members of the project team enabled the issues and uncertainties associated with collecting, analysing and interpreting the data to be discussed from different disciplinary perspectives.
