# Supplementary material for: “How long is life worth living for the horse?” A focus group study on how Austrian equine stakeholders assess quality of life for chronically ill or old horses
Source: BMC Vet Res. 2024 Aug 6;20:347. doi: 10.1186/s12917-024-04211-8 (PMC11302025; doi:10.1186/s12917-024-04211-8)
Supplement: Supplementary file 4 — Additional File 4: Code list for focus group study ‘Decisions for chronically ill and old horses’. Final categories and codes used for all focus groups with abbreviations and descriptions. [file 12917_2024_4211_MOESM4_ESM.pdf]

Code list for focus group study *'Decisions for chronically ill and old horses'*

Additional Table 2: Final categories and codes with abbreviations and descriptions.

Category / code	***Abbreviation***	***Description***
**Quality of life as a concept**	**QC**	This includes aspects that are identified as relevant to a horse's QoL.<br><br>BA - Biographical aspects: Descriptions of an individual horse's life
QC – biographical aspect – horse-based	QC-BA-HB	This includes descriptions of aspects based on the horse that are considered relevant to the horse's QoL. This includes but is not limited to basic information (such as age, breed, coat colour, size), behaviour, the mental or emotional state of the horse, health, pain, weight or body condition score, appetite, character of the horse, types and qualities of relationships with humans and with horses (and interactions within these e.g. aggressive behaviour), and preferences or what the horse dislikes.
QC – biographical aspect – resource-based	QC-BA-RMB	This includes descriptions of aspects which are considered relevant to the QoL of that horse and that are related to resources that the horse has access to and to managerial decisions by humans that shape the horse's life. This includes but is not limited to size, type and state of the horse's housing, availability and quality of food and water, the amount of time allowed feeding (e.g. at the hay rack), the type, amount and state of bedding material, the possibility for access to other horses, time allowed interacting freely with other horses, type and amount of exercise or use by humans (e.g. walking exercise based on stable layout, daily riding sessions), time allowed moving unrestrictedly on pasture (with grass) or paddock (without grass), daily routines, and the health care routine (such as frequency of check-ups by the vet or farrier visits).
**Quality of life assessment**	**QA**	This category includes aspects related to the assessment of QoL for old or chronically ill horses. QoL assessment also includes informal approaches and any less formal attempts to evaluate or judge a horse's QoL.
QA – required time	QA-TIME	This includes the specifications of the amount of time needed to assess the QoL of an old or chronically ill horse.
QA – owner	QA-OWNER	This includes specifications of the role, function and contributions of the horse owner in QoL assessments for old or chronically ill horses, including how horse owners proceed in order to arrive at an (overall) assessment of the QoL of an old or chronically ill horse and including aspects with regards to relationships with the horse owner.
QA – vets	QA-VET	This includes specifications of the role, function and contributions (such as (medical) expertise) of horse veterinarians or official veterinarians in QoL assessments for old or chronically ill horses, including how

		veterinarians proceed in order to arrive at an (overall) assessment of the QoL of an old or chronically ill horse and including aspects with regards to relationships with the veterinarian(s).
QA – others	QA-OTHER	This includes specifications of the roles, functions and contributions of other stakeholders (such as caregiver, farrier, friends and family; not included are vets and horse owners because of their individual codes) in QoL assessments for old or chronically ill horses including how these stakeholders' proceed in order to arrive at an (overall) assessment of the QoL of an old or chronically ill horse and including aspects with regards to relationships with the other stakeholders.
QA - challenges	QA-CHALL	This includes challenges and difficulties with regards to assessing QoL of old or chronically ill horses. E.g. missing information, lack of trust in quality of information, lack of objectivity due to emotional attachment, not knowing what the horse would prefer
QA - dealing with challenges	QA-DEAL	This includes identified strategies of how stakeholders deal with and attempt to overcome challenges they experience with regards to QoL assessment for old or chronically ill horses. E.g. asking a friend for an opinion, contacting a second veterinarian, imagining how they would feel in the situation the horse is in
QA – disagreement – process	QA-DIS-PRO	Participants of the group discussion explicitly disagree with each other or highlight disagreements with stakeholders outside the setting of the discussion about the requirements for, the process of, the role of stakeholders in and challenges with QoL assessments for old or chronically ill horses.
QA – disagreement – QoL of a horse	QA-DIS-HORSE	Participants of the group discussion explicitly disagree with each other or highlight disagreements with stakeholders outside the setting of the discussion about the QoL of a horse (or multiple horses) or the relevance of biographical aspects in the context of QoL of old or chronically ill horses
**Factors in decision-making**	**FD**	This category includes specifications of the role of QoL and of other factors in decision-making about veterinary interventions for old or chronically ill horses. Included in veterinary interventions are the provision of preventive, curative or palliative medicine for acute or chronic conditions, which includes euthanasia of horses but also the provision of veterinary medical expertise, for example in order to change husbandry conditions.
FD – role of QoL	FD-QOL	This includes specifications of the role and function of QoL in decision-making processes about veterinary interventions for an old or chronically ill horse.
FD – owner-related	FD-OWNER	This includes specifications of owner-related factors that influence decisions about veterinary interventions for old or chronically ill horses. These include but are not limited to owner wishes, resources available to the owner (such as money and time), the emotional bond with the horse, the intended use of the horse (such as for competition) and opinions or advice from friends and family.
FD – medical factors	FD-MEDIC	This includes specifications of medical factors that influence decisions about veterinary interventions for old or chronically ill horses. These include but are not limited to medical options and prognoses, aspects

		of the health status without considering the overall QoL, expert knowledge and the professional advice from veterinarians, attempts to advance veterinary medicine by trying new therapies.
FD – self-understanding and legal frameworks	FD-LEGAL	This includes aspects with regards to legal frameworks and the professional self-understanding of stakeholders (veterinarians, caregivers, farriers, stable but not horse owners) that influence decisions about veterinary interventions for old or chronically ill horses. E.g. an official vet referring to the treating veterinarian for a judgement on therapy options, a veterinarian highlighting the legal right of the horse owner to decide between treatment options, a farrier describing their role as focussed on the hooves of the horse and no (other) medical aspects.
FD – other factors	FD-OTHFAC	This includes specifications of the influence of other factors onto decision-making processes about veterinary interventions for an old or chronically ill horse that are neither owner-related, concerned with medical or legal aspects nor uncertainties.
FD – uncertainties	FD-UNCERT	This includes uncertainties and resulting difficulties with regards to decisions about veterinary interventions for old or chronically ill horses.
FD – disagreement – factors	FD-DIS-FAC	Participants of the group discussion explicitly disagree with each other or highlight disagreements with stakeholders outside the setting of the discussion about the role of QoL and other factors in decision-making about veterinary interventions for old or chronically ill horses.
FD – disagreement – decision	FD-DIS-DEC	Participants of the group discussion explicitly disagree with each other or highlight disagreements with stakeholders outside the setting of the discussion about a decision about a veterinary intervention for a horse (or multiple horses).
**Second Coding Cycle**		
QA – perspective changes and analogies<br><br>(Perspektivenwechsel und Analogien)	QA-PERSPECTIVE	The participant uses analogies to humans or other animals to explain how they (the participant) evaluate a particular horse's QoL or QoL of horses in general. This includes references to the participants own experiences of illness but also references to other people (the grandma) or animals (e.g. dogs).
QA – experiences and examples	QA-EXAMPLE	The participant uses a particular horse's story to illuminate, explain or justify aspects related to QoL of horses.
QA – species norm	QA-SPECIES	The participant uses the species norm as a justification for a particular horse's QoL or for the importance of aspects of horses' QoL in general. An example for this is the reference to horses typically relying on fleeing when in danger.
QA - reconsidering	QA-RECON	A participant describes changing their opinion about the QoL of a particular horse or the importance of aspects relevant for horses' QoL.
